# Unraveling the evolutionary history of the nematode *Pristionchus pacificus*: from lineage diversification to island colonization

**DOI:** 10.1002/ece3.495

**Published:** 2013-02-07

**Authors:** Angela McGaughran, Katy Morgan, Ralf J Sommer

**Affiliations:** Department for Evolutionary Biology, Max Planck Institute for Developmental BiologyTübingen, Germany

**Keywords:** Approximate Bayesian Computation, colonization, modeling, nematode, population genetics, *Pristionchus pacificus*

## Abstract

The hermaphroditic nematode *Pristionchus pacificus* is a model organism with a range of fully developed genetic tools. The species is globally widespread and highly diverse genetically, consisting of four major independent lineages (lineages A, B, C, and D). Despite its young age (∼2.1 Ma), volcanic La Réunion Island harbors all four lineages. Ecological and population genetic research studies suggest that this diversity is due to repeated independent island colonizations by *P. pacificus*. Here, we use model-based statistical methods to rigorously test hypotheses regarding the evolutionary history of *P. pacificus*. First, we employ divergence analyses to date diversification events among the four “world” lineages. Next, we examine demographic properties of a subset of four populations (“a”, “b”, “c”, and “d”), present on La Réunion Island. Finally, we use the results of the divergence and demographic analyses to inform a modeling-based approximate Bayesian computation (ABC) approach, where we test hypotheses about the order and timing of establishment of the Réunion populations. Our dating estimates place the recent common ancestor of *P. pacificus* lineages at nearly 500,000 generations past. Our demographic analysis supports recent (<150,000 generations) spatial expansion for the island populations, and our ABC approach supports c>a>b>d as the most likely colonization order of the island populations. Collectively, our study comprehensively improves previous inferences about the evolutionary history of *P. pacificus*.

## Introduction

Empirical approaches in population genetics provide a useful platform for inferring the patterns and processes underlying a species evolutionary history. However, statistical model-based approaches that actually test the inferences derived from empirical work are often under-utilized. This is unfortunate because model-based inference, such as may be derived from computer simulation, is an excellent tool for understanding the evolutionary consequences of complex processes whose interactions cannot be analytically predicted (Hoban et al. 2012).

Recent developments in population genetics have meant that specialized software previously intractable to the greater community is now an accessible option for many researchers (Hoban et al. 2012). In particular, programs exploiting approximate Bayesian computation (ABC; Beaumont et al. 2002) allow users to evaluate the fit between observed and simulated population genetic data. The likelihood-free nature of ABC removes limitations of computational intensity and allows complex evolutionary scenarios to be tested, with their evolutionary parameters (e.g. population foundation, expansion, decline and divergence, and their time of occurrence) estimated and modeled in a comparative framework (Beaumont et al. 2002; Bertorelle et al. 2010; Csilléry et al. 2010; Guillemaud et al. 2010; Hoban et al. 2012).

**Figure 1:**
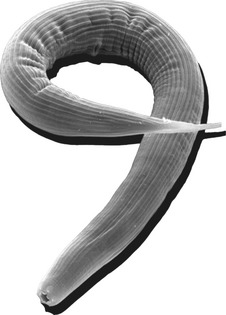
The nematode *Pristionchus pacificus*.

The hermaphroditic nematode *Pristionchus pacificus* (Fig. 1) provides a good case study for evaluation of population genetic-based inferences in a strict model-based framework. This species has been developed as a laboratory model (Hong and Sommer 2006; Sommer 2009), and an array of fully developed genetic tools, including a sequenced genome, are available (Dieterich et al. 2008). Recent integration of developmental biology with ecological and population genetics-based approaches (e.g. Mayer and Sommer 2011; Morgan et al. 2012) in *P. pacificus* allow for full exploitation of this model system from all aspects of development, genetics and ecology (e.g. Bento et al. 2010).

Extremely diverse from a genetic perspective, the *P. pacificus* gene pool consists of four distinct lineages (“A”, “B”, “C”, and “D”; Herrmann et al. 2010; Morgan et al. 2012). Assuming one generation per year, diversification between these lineages has been inferred to have occurred somewhere between 10^4^ and 10^5^ ybp, with the oldest time to most recent common ancestor (TMRCA) estimated at 1.2 × 10^6^ ybp (Molnar et al. 2011). While based on a large number of mitochondrial (mt) genetic characters (10,251 bp), this analysis incorporated just nine *P. pacificus* isolates. As such, additional divergence estimates are required to better characterize diversification in *P. pacificus*.

**Figure 2:**
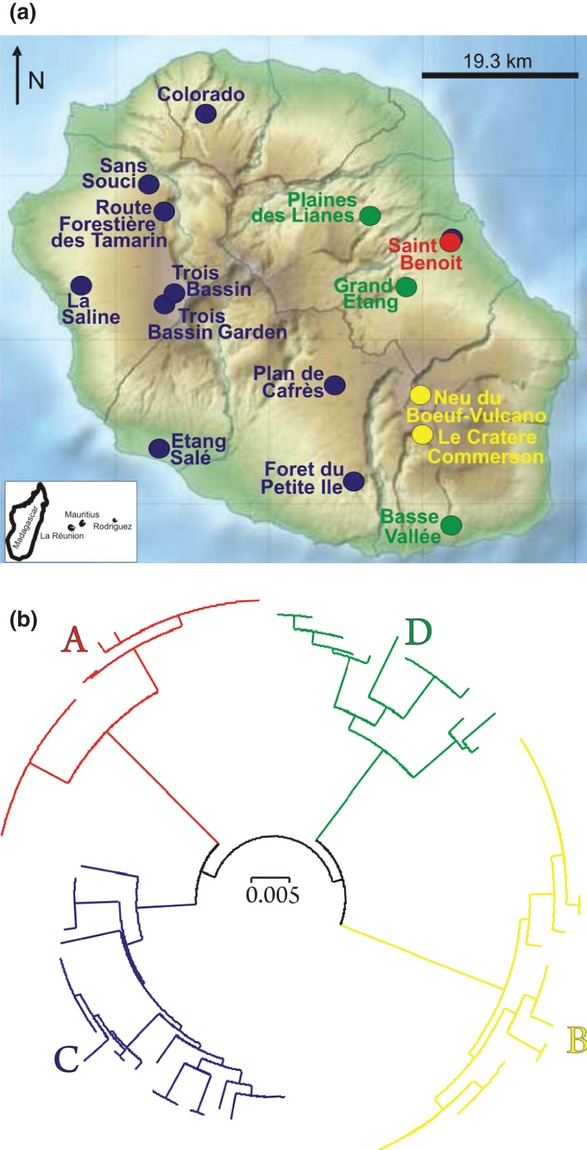
(a) Topographic map of La Réunion Island, indicating the collection localities of *Pristionchus pacificus* isolates used in this study. The four Réunion populations are indicated by the text color (population a = red, b = yellow, c = blue, d = green). The location of La Réunion Island in relation to Madagascar is indicated at the bottom left; (b) Graphic presenting the clade structure among the four selected Réunion *P. pacificus* populations (*n* = 97) based on mtDNA (see Table S1)

*P. pacificus* populations are geographically widely dispersed at the global level, but all four of its lineages are also represented in populations on La Réunion Island (Fig. 2). The young age of this island (∼2.1 Ma) makes it unlikely that lineage diversification in *P. pacificus* occurred there in situ. Indeed, ecological and population genetic research established that the presence of high diversity on La Réunion is most likely due to successful colonization events for each of the four lineages independently (Herrmann et al. 2010; Morgan et al. 2012). However, very little is known about colonization patterns on La Réunion despite their obvious importance as a component of the evolutionary history of *P. pacificus*. Population demographic components among Réunion Island *P*. *pacificus* are also currently unexplored.

Here, we use mt and microsatellite (STR) data in divergence and demographic analyses to provide lineage diversification estimates in *P. pacificus*, and to assess the nature and extent of population growth and spatial expansion among its island populations, respectively. We then apply these results in the program DIYABC to comprehensively model evolutionary history of the La Réunion *P. pacificus* populations, characterizing the timing and order of colonization events.

## Materials and Methods

### Data selection

Sequence data (565 bp) from a 760 bp mtDNA fragment (*ND6* and *ND4L* genes) and 16 STR markers representing all six *P. pacificus* chromosomes were available for several hundred strains from previous studies (GenBank/Dryad accession numbers: JN812789-JN812965/doi.10.5061/dryad.ns1stov2 for mtDNA and STR data, respectively; Herrmann et al. 2010; Morgan et al. 2012). These studies identified the presence of genetic lineages A, B, C, and D (see Introduction; Fig. 2), separated by up to 5% sequence divergence (uncorrected *P*-distance; mtDNA), and identified in neighbor-joining (Herrmann et al. 2010; mtDNA), network, and clustering analyses (Morgan et al. 2012; mtDNA and STR). Here, we worked with both a complete “world” dataset (388 sequences; lineages A, B, C, D) and a selected subset of 97 sequences representing four La Réunion populations (populations a, b, c, d; including 16–30 individuals each; Supplementary Information, Table S1). This subset was chosen to exclude strains showing any evidence for potential admixture, and to cover where possible the range of genetic diversity present in the global dataset without over-burdening our analysis (Table S1). For both datasets (“world” and “Réunion”) and both markers (mt and STR), common measures of genetic diversity (e.g. haplotype diversity, nucleotide diversity, mean number of pairwise differences, mean number of alleles, F_ST_) were calculated for each lineage/population separately in the program Arlequin ver. 3.5.1.2 (Excoffier et al. 2005).

### Divergence in *P. pacificus*

Divergence dates for the “world” mtDNA dataset of *P. pacificus* strains were estimated using BEAST ver. 1.6.1 (Drummond and Rambaut 2007). Analyses were performed over three replicates, using the nucleotide substitution model HKY+Γ with four gamma categories, as selected using jModelTest ver. 0.1 (Posada 2008). In each run, base frequencies were estimated and a strict clock model was enforced, with the rate estimated based on a uniform clock prior: 7.6 × 10^−8^ [7 × 10^−8^, 8 × 10^−8^] (see Molnar et al. 2011). The four monophyletic lineages (Herrmann et al. 2010; Morgan et al. 2012) were enforced and ‘birthdeath’ was used as a tree prior (as selected using BayesFactor tests in comparison with a Yule model; see also Molnar et al. 2011). Each MCMC analysis was run for 50,000,000 iterations (sampled every 1,000 iterations), of which the first 10% was discarded as burn-in. Convergence of the chains to a stationary distribution was confirmed by visual inspection of plotted posterior estimates in Tracer ver. 1.5 (available at http://evolve.zoo.ac.uk/software/); the effective sample size (the number of effectively independent draws from the posterior distribution to which the Markov chain is equivalent) for each parameter always exceeded 100, usually by an order of magnitude. After discarding the burn-in, log files from each set of three runs were combined using LogCombiner ver. 1.6.1 (Drummond et al. 2005), and mean node heights for each lineage were estimated along with their 95% higher posterior densities (HPD) to infer age estimates of the TMRCAs for each lineage, and overall, for the *P. pacificus* “world” strains.

### Demography of Réunion populations

To test for expansion events within the four selected Réunion populations, we compared the observed frequency distribution of pairwise nucleotide differences among individuals (i.e. mismatch distribution, MMD; Rogers and Harpending 1992) with expected distributions from a spatial population expansion using 10,000 parametric bootstrap replicates in Arlequin for the mtDNA dataset.

In this method, populations that have experienced demographic expansion generally display a unimodal distribution, while those at demographic equilibrium or in decline generally provide a multimodal distribution of pairwise differences (Slatkin and Hudson 1991; Rogers and Harpending 1992). Three parameters were estimated: θ_0_ = 2*N*_*0*_μ, θ_1_ = 2*N*_1_μ, and tau (τ) = 2μ*t*, where μ is the mutation rate for the locus. *P*-values were then calculated as the proportion of simulations producing a larger sum-of-squared deviation (SSD) than the observed SSD. The raggedness index of the observed MMD was also calculated for each of the Réunion populations and its significance determined similar to SSD as implemented in Arlequin. Small raggedness values are typical of an expanding population, whereas higher values are observed among stationary or bottlenecked populations (Harpending et al. 1993; Harpending 1994).

Values of τ were subsequently converted to time since expansion (*t*) in years before present (*t* = τ/2μ) using an mtDNA mutation estimate for *P. pacificus* (μ = 7.6 × 10^8^ substitutions per site per generation), obtained from mutation accumulation lines (Molnar et al. 2011), assuming a 1-year generation time for hermaphrodites.

### Colonization of La Réunion

Our modeling approach in the program DIYABC ver. 1.0.4.46-beta (Cornuet et al. 2008) included two events – first, lineage diversification and second, population colonization. In particular, we wished to examine the order and timing of colonization of Réunion populations, following lineage diversification. To achieve this, we described scenarios that included eight populations of mtDNA and STR data. First, lineage diversification (or, looking backward in time, *coalescence*) among lineages A–D, including 388 individuals from the “world” dataset, proceeded according to the order of diversification dating estimates provided in BEAST (D>A>C>B; see Results). Next, each lineage was modeled to undergo a split, resulting in the formation of four sub-populations (i.e. populations a–d) containing Réunion-specific strains that corresponded to the “Réunion” dataset of 97 strains; these sub-populations then ‘colonized’ the island. Island colonization was modeled by immediately following the sub-population splits with the decrease in population size (i.e. bottleneck) that necessarily accompanied their foundation – a bottleneck in DIYABC is modeled with the parameters N (effective population size), and db (duration of bottleneck); following a bottleneck event, N is reduced and stays low for the period specified by db.

Thus, our DIYABC scenarios were described by the specified parameters: lineage coalescence (with an un-sampled source population, U), occurring at times *t*_5–8_, effective population sizes for ancestral (N_A_), present day (N_1_, N_2_, N_3_, N_4_), and bottlenecked (N_5_, N_6_, N_7_, N_8_) populations, colonization of the island (divergence of sub-populations a, b, c, and d, from lineages A, B, C, and D) over a given relative order/time (*t*_1_–*t*_4_), and duration of the colonization-induced bottleneck (db). The values for given parameters were drawn from a minimum–maximum range of uniform priors (Table S2a), which means that all values bounded by the given prior are equally likely. Priors were chosen based on prior analysis (e.g. the timing of coalescence (*t*_5–8_); priors were defined as: 10,000:1,000,000 generations, based on BEAST analysis (see Results); timing of colonization priors (*t*_1–4_) were defined as 1,000:250,000 generations based on Arlequin MMD analyses (see Results). Finally, the order of diversification/colonization events was restricted to occur as *t*_8_/*t*_7_>*t*_6_, *t*_6_/*t*_5_>*t*_4_, *t*_4_>*t*_3_, *t*_3_>*t*_2_, *t*_2_>*t*_1_. Although we analyzed the scenario where lineage diversification occurred as: U>D>A>C>B, inclusion of the diversification prior *t*_8_/*t*_7_>*t*_6_, *t*_6_/*t*_5_>*t*_4_ meant that the diversification was actually free to proceed as: U>A/D>B/C (based on overlapping dating estimates in BEAST; see Results). For the STR partition of the dataset, we used default mutation model settings (e.g. a generalized stepwise mutation model, and each locus mean mutation rate drawn from a Gamma distribution with shape = 2) and we employed the Hasegawa-Kishino-Yano (HKY) mutation model (Hasegawa et al. 1985), with the number of constant sites set to 10%, and the shape of the gamma distribution for the mutation rate set to 2, for the mtDNA dataset.

For a given analysis, DIYABC first evaluates the observed dataset based on a set of selected summary statistics (see Table 1; S2b). Next, a specified number of simulations are run based on the model settings and the same summary statistics for this, the simulated dataset, are calculated. Finally, DIYABC evaluates the summary statistics of the simulated datasets for their closeness (using Euclidian distances) to those calculated in the observed dataset. The 1% of simulated datasets producing summary statistics closest to the observed summary statistics can then be used to estimate the relative posterior probability of each scenario via a logistic regression estimate (Cornuet et al. 2008), and principal components analysis (PCA) can be applied to visualize how similar the simulated and observed statistics are for each scenario. Using the logistic regression (taking the highest posterior probability as indicating the most likely scenario) and PCA estimates, the most likely scenario in the analysis can be selected.

**Table 1:** Table of various summary statistics generated in Arlequin for both STR and mt, and for “world” and “Réunion” datasets (see Methods). A subset of these statistics were used in DIYABC to compare simulated with observed datasets

	“World” lineages				“Réunion” populations			
		
Statistic	A	B	C	D	A	B	C	D
mtDNA								
No. of gene copies	58	29	163	33	22	29	30	16
No. of haplotypes	37	12	43	21	8	12	16	10
No. of polymorphic sites	91	22	72	57	15	22	40	30
Haplotype diversity (S.D.)	0.943 (0.023)	0.882 (0.038)	0.899 (0.014)	0.945 (0.025)	0.758 (0.078)	0.882 (0.038)	0.924 (0.031)	0.917 (0.049)
Nucleotide diversity (S.D.)	0.019 (0.009)	0.007 (0.004)	0.011 (0.006)	0.016 (0.008)	0.008 (0.004)	0.007 (0.004)	0.011 (0.006)	0.014 (0.007)
Mean no. of pairwise differences (S.D.)	13.672 (6.230)	5.039 (2.520)	8.153 (3.802)	11.949 (5.545)	5.982 (2.965)	5.039 (2.520)	7.834 (3.751)	10.033 (4.843)
Tajima's D	−1.060	−0.356	−1.110	−0.551	1.637	−0.356	−0.825	0.454
*P*-value	(0.122)	(0.397)	(0.134)	(0.328)	(0.957)	(0.404)	(0.208)	(0.721)
Mean no. of alleles (S.D.)	1.132 (0.362)	1.030 (0.170)	1.106 (0.333)	1.080 (0.282)	1.022 (0.155)	1.030 (0.170)	1.058 (0.251)	1.042 (0.208)
F_ST_*								
-B	*0.754*				*0.876*			
-C	*0.695*	*0.787*	*–*	*–*	*0.784*	*0.815*	*–*	
-D	*0.674*	*0.761*	*0.731*	*–*	*0.811*	*0.808*	*0.748*	
STR								
No. of gene copies	116	58	326	66	44	58	60	32
No. of haplotypes	56	23	149	31	21	22	29	14
No. of polymorphic loci	14	14	10	11	15	13	11	13
Mean no. of alleles (S.D.)	11.421 (3.322)	3.053 (1.580)	11.632 (10.683)	7.737 (3.263)	3.062 (1.237)	3.375 (1.500)	7.250 (4.669)	3.812 (2.228)
Mean allelic range (S.D.)	37.474 (21.054)	9.471 (7.787)	23.667 (23.607)	21.211 (23.129)	8.188 (9.669)	10.000 (7.720)	18.938 (20.352)	9.812 (10.426)
Garza-Williamson statistic (S.D.)	0.395 (0.226)	0.518 (0.335)	0.556 (0.181)	0.583 (0.305)	0.608 (0.337)	0.488 (0.321)	0.573 (0.301)	0.508 (0.260)
Mean genic diversity (S.D.)	0.707 (0.361)	0.415 (0.308)	0.438 (0.240)	0.562 (0.300)	0.343 (0.188)	0.383 (0.210)	0.517 (0.279)	0.480 (0.260)
Expected heterozygosity (S.D.)	0.741 (0.132)	0.371 (0.318)	0.563 (0.315)	0.633 (0.214)	0.362 (0.266)	0.425 (0.315)	0.638 (0.281)	0.490 (0.228)
F_ST_*								
-B	*0.163*	*–*	*–*	*–*	*0.795*	*–*	*–*	*–*
-C	*0.562*	*0.680*			*0.694*	*0.772*	*–*	*–*
-D	*0.078*	*0.321*	*0.515*		*0.556*	*0.806*	*0.533*	
Δμ^2^								
-B	90.618	–	–	–	242.931	–	–	–
-C	90.635	187.302	–	–	242.086	209.792	–	–
-D	14.416	125.063	73.209	–	58.074	177.643	106.579	–

* Significance indicated with italics

Due to the high computational load, we evaluated the 24 possible colonization orders (see Fig. S1) over two runs (each containing 12 scenarios with 250,000 simulations each), and then tested the top six most likely scenarios in a final analysis. The overall most likely scenario (based on logistic regression and PCA) was then evaluated under each of the quality control-based options in DIYABC including pre-evaluation of scenario-prior combinations, model checking, computation of bias and precision, and evaluation of confidence in scenario choice. Values of the posterior probabilities of *t*_5–8_, the time of coalescence, and *t*_1–4_, the timing of colonizations, were then estimated and compared with BEAST- and/or MMD-generated values.

## Results

### Divergence in *P. pacificus*

Analysis of divergence using the mtDNA “world” dataset in BEAST found that the mean dates to TMRCA of the four *P. pacificus* lineages ranged from 72,000 (lineage B) to 237,000 (lineage D) generations, with the mean root height (i.e. age) of the tree approximating 465,000 generations [349,000:593,000] (Table 2a). The corresponding lineage diversification order was: D>A>C>B.

**Table 2:** (a) Results from diversification analyses performed in BEAST for the four *Pristionchus pacificus* lineages, A, B, C, and D, using the mtDNA “world” dataset. Time estimates to the most recent common ancestor (TMRCA) overall, and for the four lineages are provided in generations. Estimated values for TMRCAs are rounded to the nearest 10^3^; (b) Results from mismatch distribution analysis performed in Arlequin for the four *Pristionchus pacificus* La Réunion populations, a, b, c and d, using the mtDNA “Réunion” dataset. “*t*” represents time since expansion in years before present (ybp) based on the relationship between τ and *t* (*t* = τ/2μ) assuming a 1-year generation time and a mutation rate estimate of 4.294 × 10^−5^ substitutions per locus per year; values for *t* are rounded to the nearest 10^3^; (c) Mean, median, and 25 and 75% posterior quartiles of the parameters estimated in DIYABC runs using both STR and mtDNA data for the most likely diversification/colonization scenario (see Results). Presented parameters (i.e. *t*_8_–*t*_1_, moving forward in time) correspond to the order/timing of lineage diversification (*t*_5–8_) and island colonization (*t*_1–4_), U>D/A>C>B>c>a>b>d. All parameter estimate values are rounded to the nearest 10^3^. See Methods and Results for further information

(a) Summary statistic	TMRCA(all)	TMRCA(A)	TMRCA(B)	TMRCA(C)	TMRCA(D)
Mean	465,000	191,000	72,000	133,000	237,000
Standard error of mean	1,148	711	566	702	903
Median	458,000	189,000	70,000	130,000	234,000
Geometric mean	461,000	189,000	70,000	131,000	234,000
95% HPD lower	349,000	144,000	39,000	85,000	162,000
95% HPD upper	593,000	241,000	109,000	183,000	314,000

(b) Réunion population	τ(mean)	t(ybp)
a	7.498(11.231)	87,000
b	5.030(5.672)	59,000
c	7.967(7.826)	93,000
d	10.718(11.520)	125,000

(c) Parameter	Mean	Median	q25	q75
*t*_1_	133,000	136,000	97,000	161,000
*t*_2_	158,000	156,000	117,000	187,000
*t*_3_	178,000	176,000	137,000	207,000
*t*_4_	191,000	201,000	164,000	227,000
*t*_5_	391,000	364,000	268,000	489,000
*t*_6_	585,000	587,000	384,000	787,000
*t*_7_	691,000	712,000	539,000	864,000
*t*_8_	689,000	709,000	547,000	858,000

### Demography of Réunion populations

In the MMD analyses using the mtDNA “Réunion” dataset, unimodal distributions consistent with the spatial expansion model were recovered for Réunion populations b, c, and d, while SSD and raggedness values were generally consistent with the spatial expansion model for all populations (Fig. S2). Dating analyses using the τ parameter suggested that the expansion events occurred from 59,000 (population b) to 125,000 (population d) ybp (τ values ranged from 5.03 to 10.72; Table 2b).

### Colonization of La Réunion

Lineage diversification and population colonization were modeled in DIYABC (with both STR and mtDNA “world” and “Réunion” datasets) to establish the order and timing of evolutionary events in *P. pacificus* (see Fig. S1). The lineage diversification order was set to U>A/D>B/C, based on BEAST results, and a total of 24 colonization scenarios were tested.

**Figure 3:**
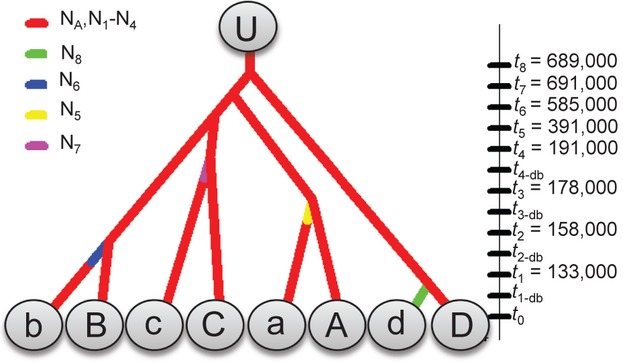
Graphic to show the most likely DIYABC lineage diversification/island colonization scenario for genetic lineages and populations of *Pristionchus pacificus*, based on STR and mt markers. In the final analysis, the overall best scenario (of 24 possibilities; see Fig. S1) corresponded to a lineage diversification (lineages A, B, C, and D, diverging away from an unsampled source population, “U”) order of: U>D/A>C>B at times *t*_5_–*t*_8_, and an island colonization (sub-populations a, b, c, and d, diverging away from their respective lineages) order of: c>a>b>d, occurring at times *t*_1_–*t*_4_. The island colonization was modeled by following the divergence of the island population away from the ancestral lineage with an immediate decrease in population size (i.e. a foundation bottleneck). The bottleneck is represented in the graphic as a magenta break upon the colonization arrows, and the bottleneck duration is the same for each population (db = 5 generations). In the graphic, the time axis is to relative scale only. Refer to Methods and Results for further information.

For the overall most likely scenario (based on logistic regression and PCA) in DIYABC, parameter values aligned well with BEAST and MMD results, and most observed summary statistics were in the range of simulated ones (Fig. S3). The parameters *t*_5_–*t*_8_, which represent the order/relative timing of lineage diversification, ranged from 391,000 to 691,000 generations, corresponding well with the relative BEAST estimates (Fig. 3; Table 2), although absolute values were approximately two- to threefold higher. The most likely colonization scenario corresponded to the population order: c>a>b>d (posterior probability: 1.000). Estimated dates for colonization (*t*_1–4_) ranged from 133,000 to 191,000 generations, corresponding well with the relevant MMD estimates (Table 2). Thus, our final estimate of the evolutionary history of *P. pacificus* corresponds to: U>D/A>C>B>c>a>b>d (Fig. 3).

## Discussion

We have comprehensively investigated the evolutionary history of hermaphroditic *P. pacificus* nematodes, with a special focus on modeling lineage diversification and island colonization. Three major conclusions can be drawn from our work, providing insight into *P. pacificus* lineage diversification, and population foundation and expansion.

Multiple mt-based divergence dating estimates suggest that diversification occurred early in the evolutionary history of *P. pacificus* lineages. BEAST analysis estimated TMRCAs of the four lineages ranging from 72,000 to 237,000 generations, with an overall root age of 465,000 [349,000–593,000] generations. In codon-partitioned dating analyses using the same program and parameter settings but a subset of nine isolates Molnar et al. (2011) estimated TMRCAs among lineages at 280,000–560,000 generations, with an overall root age of 1,120,000 [1,100,000–1,300,000] generations. Thus, our BEAST estimates fall within the confidence intervals, but are roughly one-third to one half lower than the estimates of Molnar et al. (2011). However, our DIYABC ‘*t*’ estimates (representing coalescence of *P. pacificus* lineages) ranged from 391,000 generations (diversification of C and B lineages) to 691,000 generations (diversification of A and D lineages), thus agree well with the estimates of Molnar et al. (2011). Our dating calculations all rely on a 1-year generation time estimate for *P. pacificus*. Because accurate characterization of generation times outside the laboratory is problematic, care should be taken when interpreting these dating estimates (see also Molnar et al. 2011). However, accounting for differences in strain selection and number of genetic characters between the two studies (Molnar et al. analyzed up to 10,251 bp for 9 strains, while we analyze 565 bp and 16 STR loci in up to 388 strains), it is clear that divergence in the *P. pacificus* meta-population most likely preceded the colonization of La Réunion Island.

Signals of population expansion detected in the demographic (MMD; mt-based) analyses indicated that all four selected Réunion populations have undergone spatial expansion in the period 59,000–125,000 ybp. These dates also correspond well with our colonization date estimates, generated in DIYABC (i.e. *t*_1–4_), which ranged from 133,000 to 191,000 generations, suggesting that population growth may have rapidly succeeded foundation of the four island populations. Collectively, these demographic estimates suggest that populations can undergo rapid increases in local effective population size after contraction events, pointing to the potential success of *P. pacificus*, and perhaps androdioecious species in general, in recovering population size following disturbance.

Our DIYABC analyses using both STR and mt markers supported a scenario in which the colonization of Réunion populations proceeded as: c>a>b>d. Colonization episodes appear to have occurred in the narrow time range corresponding to: 133,000–191,000 generations past. However, small but significant differences in both the relative timing and location of these colonization events may explain present differences in distribution and differentiation among the island populations (see Morgan et al. 2012). For example, we identified the most likely invasion order to begin with population c, which may suggest that early colonization of La Réunion allowed for the consequent structure and widespread western distribution observed in this population today. Conversely, more recent eastern colonization by populations b and d has presumably left less time for their dispersal to other geographic regions. Population a, despite an inferred early colonization (178,000 generations ago), is also more narrowly dispersed across its eastern distribution (see Morgan et al. 2012). Thus, it may be that establishment in eastern regions of the island is subject to greater dispersal restriction subsequent to foundation.

Colonization order may also have been important in terms of defining niche exclusivity across distinct La Réunion ‘ecozones’ (see also Morgan et al. 2012). At Saint Benoit, for example, sympatric a and d populations exist, but appear to remain genetically isolated. Isolation among populations following differentially timed foundation may have resulted in a suite of phenotypic and genotypic differences as locally isolated groups diverge in adaptive traits and/or host specificity. Defining natural variation in phenotypic traits in an evolutionary context is an on-going aim of our research group (Hong et al. 2008; Bento et al. 2010; Mayer and Sommer 2011).

## Conclusions and future work

Ultimately, we have traced the evolutionary history of *P. pacificus*, from its early diversification through to its colonization of La Réunion Island. An important avenue for future research will be to examine how far the apparently discrete island populations are characterized by genetic admixture and whether they also show degrees of adaptive phenotypic variation. In addition, better understanding of the worldwide genetic structure and diversification of *P. pacificus*, which can only be provided by extensive future biogeographic sampling of additional territories, will help improve future modeling-based studies.

## Supporting Information

**Table S1**. List of the *Pristionchus pacificus* samples used in the “Réunion” analyses of this study, and their relevant collection information, including sampling location, host beetle species, and population code (based on mtDNA and STR data from 16 loci). See Herrmann et al. 2010 and Morgan et al. 2012 for additional strain information about the “world” dataset strains.

**Figure S1**. Graphic to demonstrate the 24 colonization scenarios tested in DIYABC using STR and mt markers; our models examined all possible island colonization orders following the lineage diversification (lineages A, B, C, and D, diverging away from an unsampled source population, "U") order U>D>A>C>B. Island colonization (i.e. sub-populations a, b, c, and d, diverging away from their respective lineages) was modeled by following the divergence of the island population away from the ancestral lineage with an immediate decrease in population size (i.e. a foundation bottleneck). In the presented examples, lineage diversification proceeds through times *t*_5_−*t*_8_, and is followed by colonization of populations at times *t*_1_−*t*_4_; colonization orders in the figure are presented with a, b, c, and d first for (a), (b), (c), and (d). In each case, the bottleneck is represented as population size changes (colored bars in figure; N_5_, N_6_, N_7_, and N_8_), and the bottle-neck duration is the same for each population (db = 5 generations). The time axis is to relative scale only. Refer to Methods and Results for further information.

**Figure S2**. Observed distribution of pairwise differences (i.e. MMD) between mt haplotypes in the four selected populations (a, b, c, d, corresponding to (a), (b), (c), and (d) in the figure) of *Pristionchus pacificus* on La Réunion Island. Expected distributions were calculated both numerically (i.e. observed data; dark blue bars in figure) and with simulated data (light blue lines in figure) using mtDNA in Arlequin. The observed (unimodal) distributions for populations b, c, and d are consistent with the spatial expansion model, while the SSD and raggedness values given above each distribution plot are consistent with the spatial expansion model for all populations.

**Figure S3**. Principal component analysis (PCA) of the 1% of simulated datasets generated in DIYABC that were closed to the observed dataset in terms of summary statistics (see Methods) for the most likely colonization scenarios (*n* = 6) in analyses using mt and STR markers for *P. pacificus*, showing that most observed summary statistics fall within the range of simulated ones. Initial tests examined all possible orders (*n* = 24) of island colonization under the lineage diversification scenario U>D/A>C>B, and subsequent analysis considered the six most likely scenarios (see Table S3). The final most likely colonization scenario (logistic regression value: 1.000) was: c>a>b>d. See Results for further information.
