# Supplementary material for: Unraveling the evolutionary history of the nematode Pristionchus pacificus: from lineage diversification to island colonization
Source: Ecol Evol. 2013 Feb 7;3(3):667–75. doi: 10.1002/ece3.495 (PMC3605854; doi:10.1002/ece3.495)

**Electronic Supplementary Material (ESM)**

Table S1. List of the *Pristionchus pacificus* samples used in the ‘Réunion’ analyses of this study, and their relevant collection information, including sampling location, host beetle species and population code (based on mtDNA and STR data from 16 loci). See Herrmann *et al.,* 2010 and Morgan *et al.,* 2012 for additional strain information about the ‘world’ dataset strains.

sample location host beetle species population code

RS5413 Saint Benoit *Adoretus* sp. a

RS5414 Saint Benoit *Maladera affinis* a

RS5416 Saint Benoit *Maladera affinis* a

RS5417 Saint Benoit *Maladera affinis* a

RS5420 Saint Benoit *Maladera affinis* a

RS5421 Saint Benoit *Maladera affinis* a

RS5422 Saint Benoit *Maladera affinis* a

RS5423 Saint Benoit *Maladera affinis* a

RS5424 Saint Benoit *Maladera affinis* a

RSB068 Saint Benoit *Aphodius* sp. a

RSB069 Saint Benoit *Aphodius* sp. a

RSB070 Saint Benoit *Aphodius* sp. a

RSB071 Saint Benoit *Aphodius* sp. a

RSB077 Saint Benoit *Aphodius* sp. a

RSB078 Saint Benoit *Aphodius* sp. a

RSB079 Saint Benoit *Aphodius* sp. a

RSB080 Saint Benoit *Aphodius* sp. a

RSB081 Saint Benoit *Maladera affinis* a

RSB082 Saint Benoit *Maladera affinis* a

RSB084 Saint Benoit *Maladera affinis* a

RSB085 Saint Benoit *Maladera affinis* a

RSB086 Saint Benoit *Aphodius* sp. a

RS5361 Neu du Boeuf-Vulcano Soil b

RSA075 Neu du Boeuf-Vulcano *Amneidus godefroyi* b

RSA076 Neu du Boeuf-Vulcano *Amneidus godefroyi* b

RSB001 Le Cratere Commerson *Amneidus godefroyi* b

RSB002 Le Cratere Commerson *Amneidus godefroyi* b

RSB003 Le Cratere Commerson *Amneidus godefroyi* b

RSB004 Le Cratere Commerson *Amneidus godefroyi* b

RSB005 Le Cratere Commerson *Amneidus godefroyi* b

RSB006 Le Cratere Commerson *Amneidus godefroyi* b

RSB007 Le Cratere Commerson *Amneidus godefroyi* b

RSB008 Le Cratere Commerson *Amneidus godefroyi* b

RSB009 Le Cratere Commerson *Amneidus godefroyi* b

RSB010 Le Cratere Commerson *Amneidus godefroyi* b

RSB011 Le Cratere Commerson *Amneidus godefroyi* b

RSB012 Le Cratere Commerson *Amneidus godefroyi* b

RSB013 Le Cratere Commerson *Amneidus godefroyi* b

RSB014 Le Cratere Commerson *Amneidus godefroyi* b

RSB015 Le Cratere Commerson *Amneidus godefroyi* b

RSB016 Le Cratere Commerson *Amneidus godefroyi* b

RSB033 Neu du Boeuf-Vulcano *Amneidus godefroyi* b

RSB034 Neu du Boeuf-Vulcano *Amneidus godefroyi* b

RSB035 Neu du Boeuf-Vulcano *Amneidus godefroyi* b

RSB036 Neu du Boeuf-Vulcano *Amneidus godefroyi* b

RSB037 Neu du Boeuf-Vulcano *Amneidus godefroyi* b

RSB038 Neu du Boeuf-Vulcano *Marronus borbonicus* b

RSB039 Neu du Boeuf-Vulcano *Marronus borbonicus* b

RSB040 Neu du Boeuf-Vulcano *Marronus borbonicus* b

RSB041 Neu du Boeuf-Vulcano *Marronus borbonicus* b

RSB042 Neu du Boeuf-Vulcano *Marronus borbonicus* b

RS5347 Trois Bassin *Oryctes borbonicus* c

RS5350 Etang Salé *Hoplia retusa* c

RS5394 Trois Bassin *Oryctes borbonicus* c

RS5397 Trois Bassin *Oryctes borbonicus* c

RS5399 Trois Bassin *Oryctes borbonicus* c

RS5403 Trois Bassin *Hoplia retusa* c

RS5405 Trois Bassin *Hoplia retusa* c

RS5429 Trois Bassin Garden *Hoplochelus* sp. c

RS5431 Trois Bassin Garden *Maladera affinis* c

RSA011 Trois Bassin *Oryctes borbonicus* c

RSA018 Trois Bassin *Oryctes borbonicus* c

RSA067 Trois Bassin *Hoplochelus* sp. c

RSA072 Trois Bassin *Hoplochelus* sp. c

RSA085 Plan de Cafrès *Hoplia retusa*  c

RSA086 Plan de Cafrès *Hoplia retusa* c

RSA092 Trois Bassin *Hoplia retusa* c

RSA094 Trois Bassin *Hoplia retusa* c

RSA103 Sans Souci *Oryctes borbonicus* c

RSA110 Sans Souci *Oryctes borbonicus* c

RSB018 Colorado *Adoretus* sp. c

RSB019 Colorado *Adoretus* sp. c

RSB021 Colorado *Adoretus* sp. c

RSB048 Plan de Cafrès *Hoplia retusa* c

RSB052 Plan de Cafrès *Hoplia retusa* c

RSB056 Plan de Cafrès *Hoplia retusa* c

RSB067 Foret du Petite Ile *Adoretus* sp. c

RSB072 Saint Benoit *Aphodius* sp. c

RSB074 Saint Benoit *Aphodius* sp. c

RSB091 Trois Bassin *Hoplochelus* sp. c

RSB096 Trois Bassin *Hoplia retusa* c

RS5342 Basse Vallée *Adoretus* sp. d

RS5406 Basse Vallée *Adoretus* sp. d

RSA038 Basse Vallée *Adoretus* sp. d

RSA039 Basse Vallée *Adoretus* sp. d

RSA040 Basse Vallée *Adoretus* sp. d

RSA044 Basse Vallée *Adoretus* sp. d

RSA047 Grand Etang *Adoretus* sp. d

RSA048 Grand Etang *Adoretus* sp. d

RSA050 Grand Etang *Adoretus* sp. d

RSA056 Grand Etang *Adoretus* sp. d

RSA057 Grand Etang *Adoretus* sp. d

RSB061 Plaines des Lianes *Adoretus* sp. d

RSB062 Plaines des Lianes *Adoretus* sp. d

RSB063 Plaines des Lianes *Adoretus* sp. d

RSB064 Plaines des Lianes *Adoretus* sp. d

RSB065 Plaines des Lianes *Adoretus* sp. d

Table S2. (a) Minimum-maximum range of priors used in DIYABC; (b) Summary statistics used to evaluate DIYABC simulations in the context of the observed data. See Methods for further information.

(a)

parameter prior distribution

N_A_ Uniform[10:100,000]

*t*_1_ Uniform[1,000:250,000;]

*t*_2_ Uniform[1,000: 250,000; > *t*_1_]

*t*_3_ Uniform[1,000: 250,000; > *t*_2_]

*t*_4_ Uniform[1,000: 250,000; > *t*_3_]

*t*_5_ Uniform[10,000:1,000,000; > *t*_4_]

*t*_6_ Uniform[10,000:1,000,000; > *t*_4_]

*t*_7_ Uniform[10,000:1,000,000; > *t*_6_]

*t*_8_ Uniform[10,000:1,000,000; > *t*_6_]

db Uniform[5:5]

N_1_ Uniform[10:100,000]

N_2_ Uniform[10:100,000]

N_3_ Uniform[10:100,000]

N_4_ Uniform[10:100,000]

N_5_ Uniform[1:100]

N_6_ Uniform[1:100]

N_7_ Uniform[1:100]

N_8_ Uniform[1:100]

(b)

marker 1-sample statistics 2-sample statistics

mtDNA Number of haplotypes Number of haplotypes

Number of segregating sites Number of segregating sites

Mean of pairwise differences Mean of pairwise differences (W)

Variance of pairwise differences Mean of pairwise differences (B)

Tajima’s D F_ST_

STR Mean number of alleles Mean number of alleles

Mean genic diversity Mean genic diversity

Mean size variance Mean size variance

Mean Garza-Williamson’s M F_ST_

Δµ^2^ distance

Table S3. The various DIYABC analyses performed using mt and STR markers for *P. pacificus*. Tests examined 24 possible orders of island colonisation using the ‘world’ plus ‘Réunion’ datasets, under the most likely lineage diversification order (U>D/A>C>B), in two runs consisting of 12 scenarios each (run no. 1 and 2). The most likely scenarios from runs 1 and 2 (shown here in bold text), as selected based on logistic regression and PCA in DIYABC (and in all cases, the most likely scenarios represented those with the highest regression scores; see Methods), were then evaluated in a final analysis, the posterior probabilities for which are given in square brackets in the table. Thus, the overall most likely colonisation scenario, for which the posterior probability = 1.000, was: U>D/A>C>B>c>a>b>d. See Results for further information.

Run no. Scenario Run no. Scenario

1 U>D>A>C>B>a>b>c>d 2 **U>D>A>C>B>c>a>b>d [1.000]**

U>D>A>C>B>a>b>d>c U>D>A>C>B>c>a>d>b

U>D>A>C>B>a>c>b>d U>D>A>C>B>c>b>a>d

**U>D>A>C>B>a>c>d>b [0.000]** U>D>A>C>B>c>b>d>a

**U>D>A>C>B>a>d>b>c [0.000]** U>D>A>C>B>c>d>a>b

U>D>A>C>B>a>d>c>b **U>D>A>C>B>c>d>b>a [0.000]**

U>D>A>C>B>b>a>b>d **U>D>A>C>B>d>a>b>c [0.000]**

U>D>A>C>B>b>a>d>b U>D>A>C>B>d>a>c>b

U>D>A>C>B>b>c>a>d U>D>A>C>B>d>b>a>c

U>D>A>C>B>b>c>d>a **U>D>A>C>B>d>b>c>a [0.000]**

U>D>A>C>B>b>d>a>c U>D>A>C>B>d>c>a>b

U>D>A>C>B>b>d>c>a U>D>A>C>B>d>c>b>a

Figure S1. Graphic to demonstrate the 24 colonisation scenarios tested in DIYABC using STR and mt markers; our models examined all possible island colonisation orders following the lineage diversification (lineages A, B, C and D, diverging away from an unsampled source population, ‘U’) order U>D>A>C>B. Island colonisation (i.e. sub-populations a, b, c and d, diverging away from their respective lineages) was modelled by following the divergence of the island population away from the ancestral lineage with an immediate decrease in population size (i.e. a foundation bottleneck). In the presented examples, lineage diversification proceeds through times *t*_5_-*t*_8_, and is followed by colonisation of populations at times *t*_1_-*t*_4_; colonisation orders in the figure are presented with a, b, c and d first for (a), (b), (c) and (d). In each case, the bottleneck is represented as population size changes (coloured bars in figure; N_5_, N_6_, N_7_ and N_8_), and the bottleneck duration is the same for each population (db = 5 generations). The time axis is to relative scale only. Refer to Methods and Results for further information.


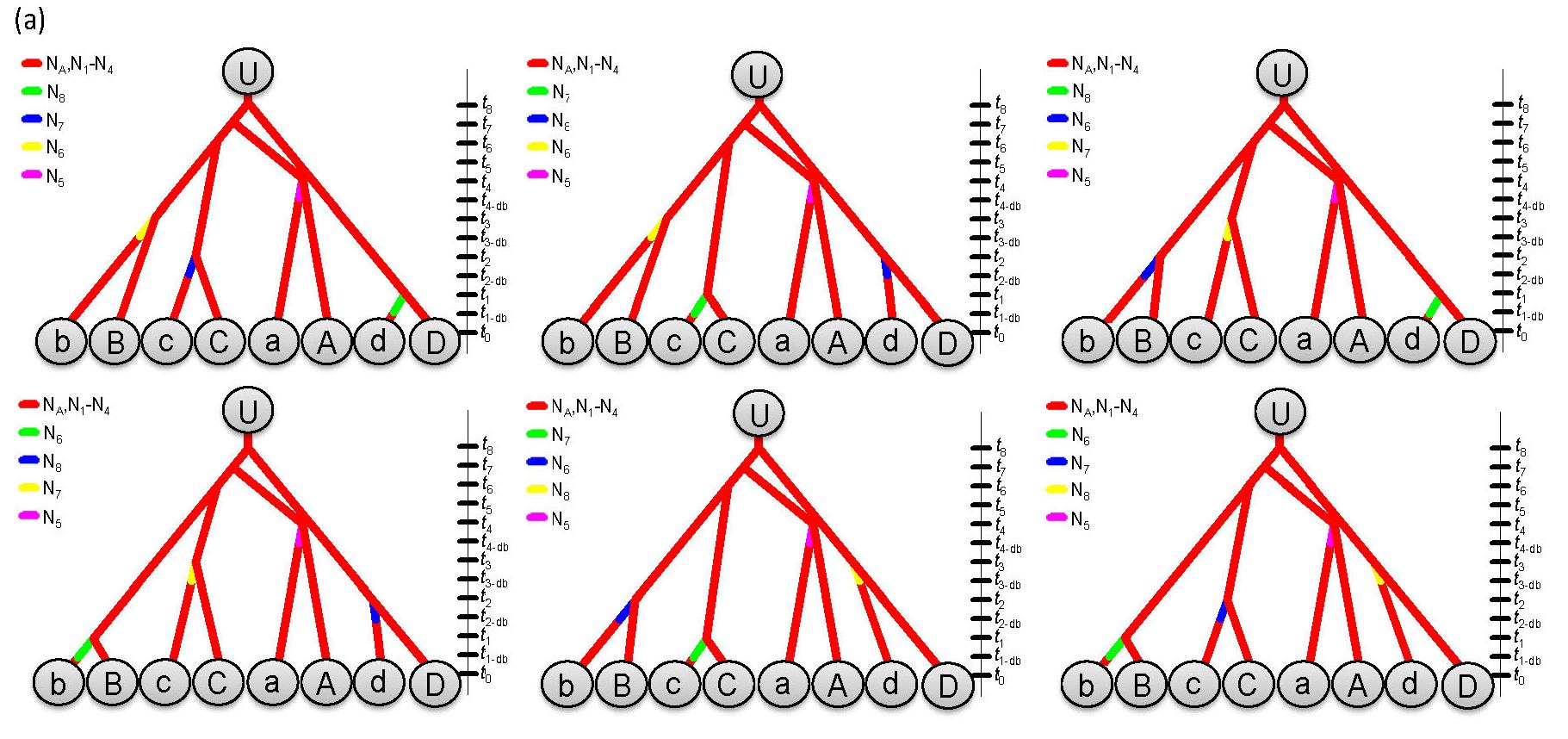

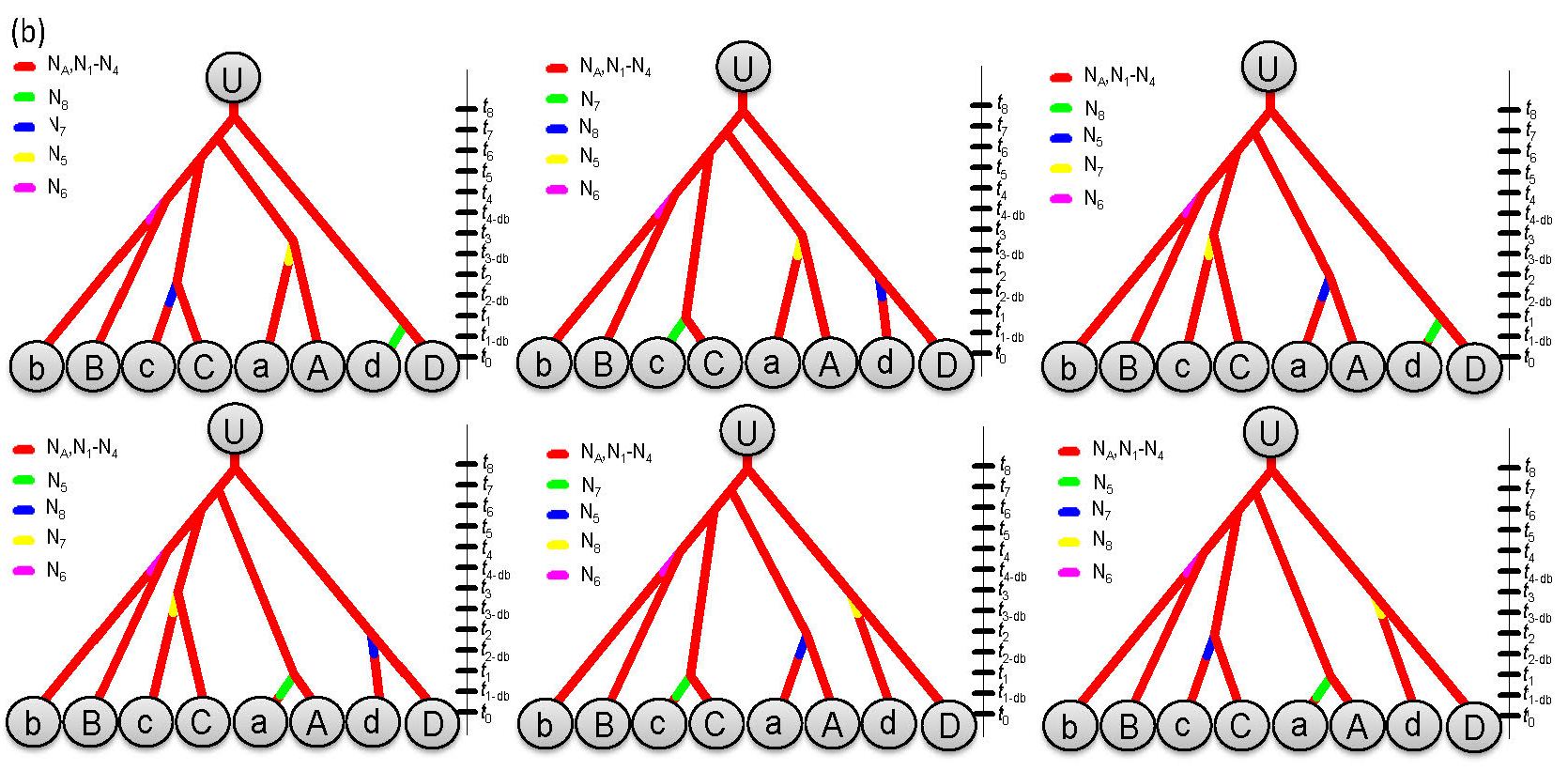

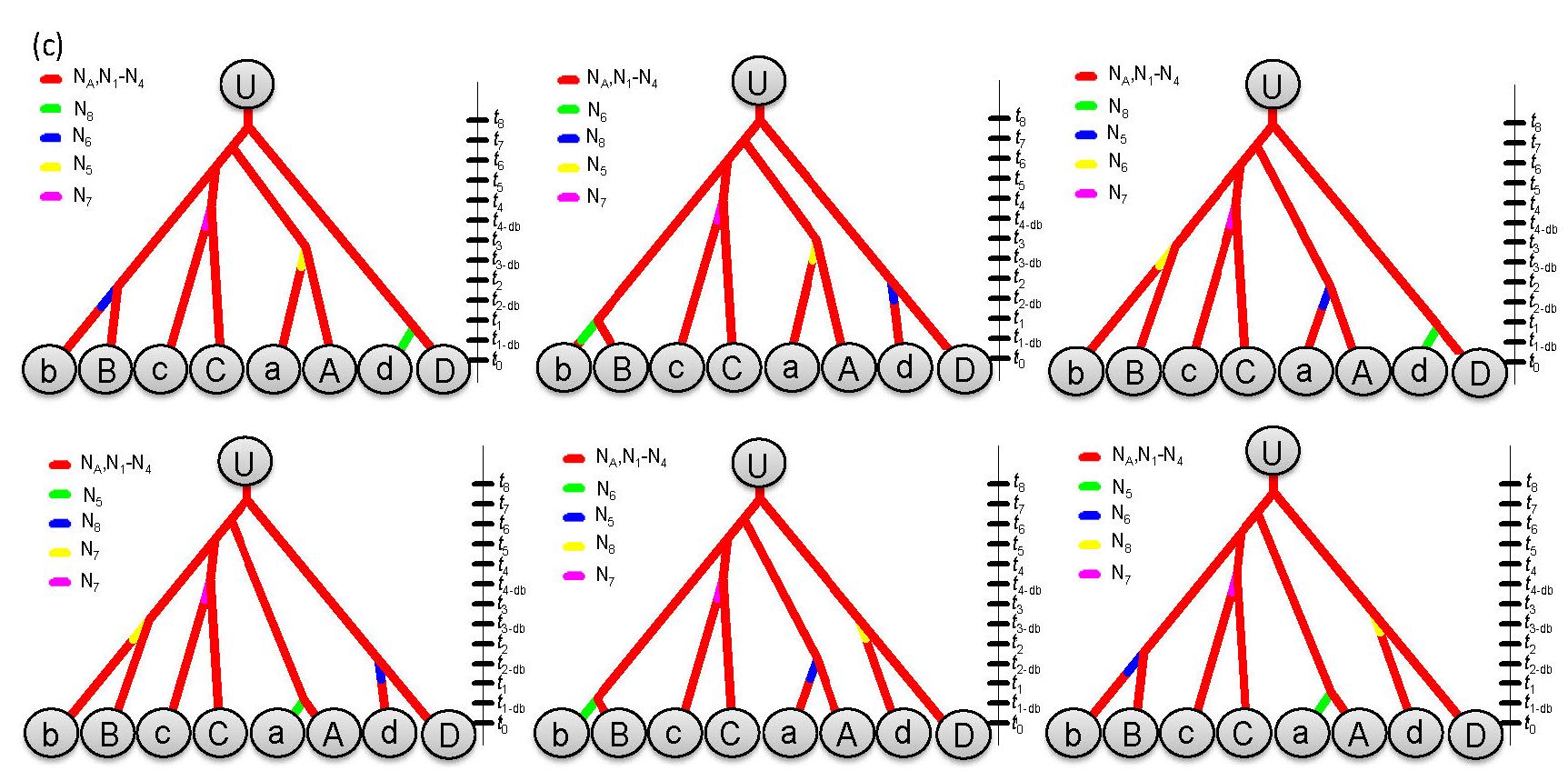


Figure S2. Observed distribution of pairwise differences (i.e. MMD) between mt haplotypes in the four selected populations (a, b, c, d, corresponding to (a), (b), (c), and (d) in the figure) of *Pristionchus pacificus* on La Réunion Island. Expected distributions were calculated both numerically (i.e. observed data; dark blue bars in figure) and with simulated data (light blue lines in figure) using mtDNA in Arlequin. The observed (unimodal) distributions for populations b, c, and d are consistent with the spatial expansion model, while the SSD and raggedness values given above each distribution plot are consistent with the spatial expansion model for all populations.


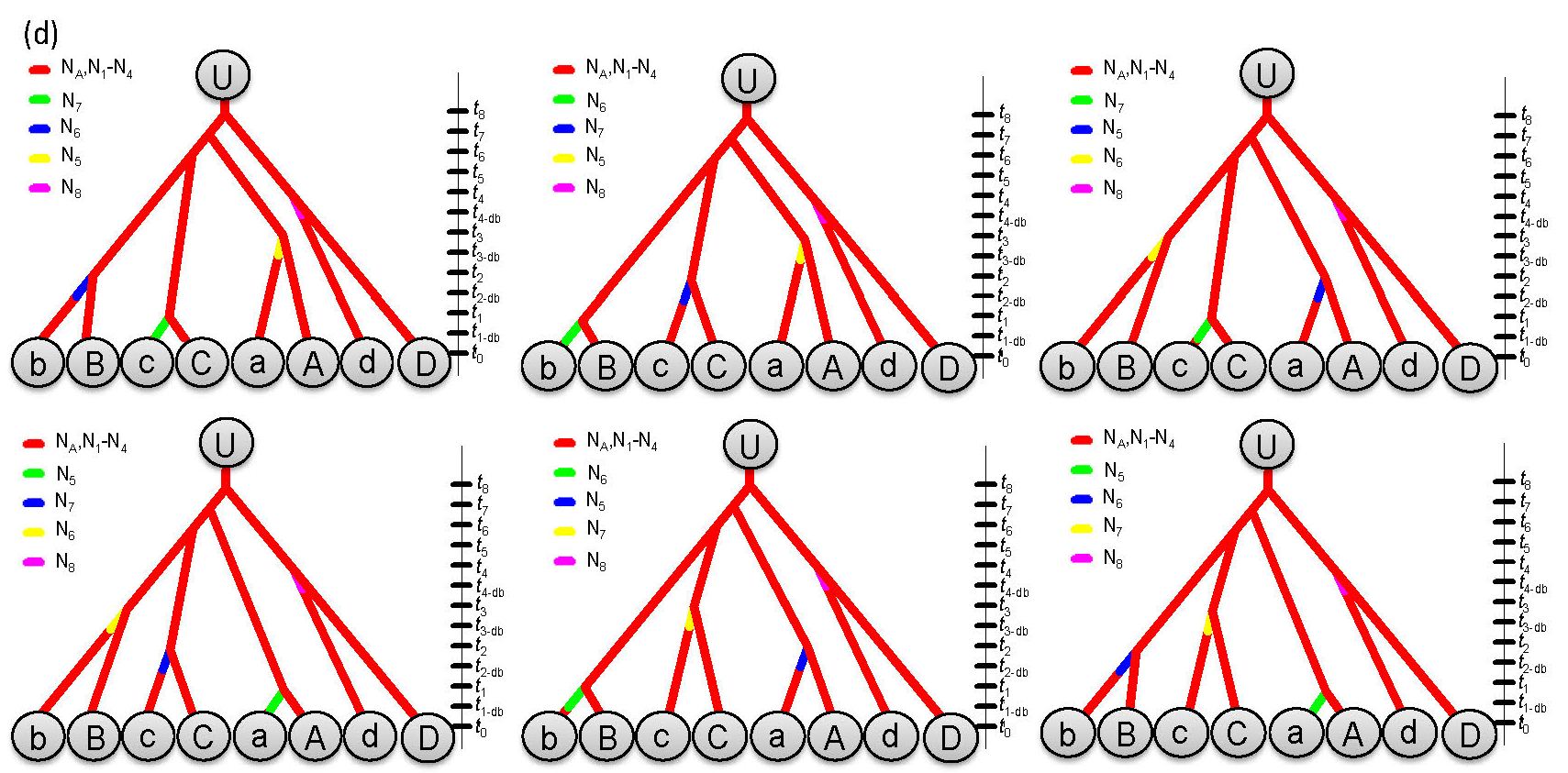

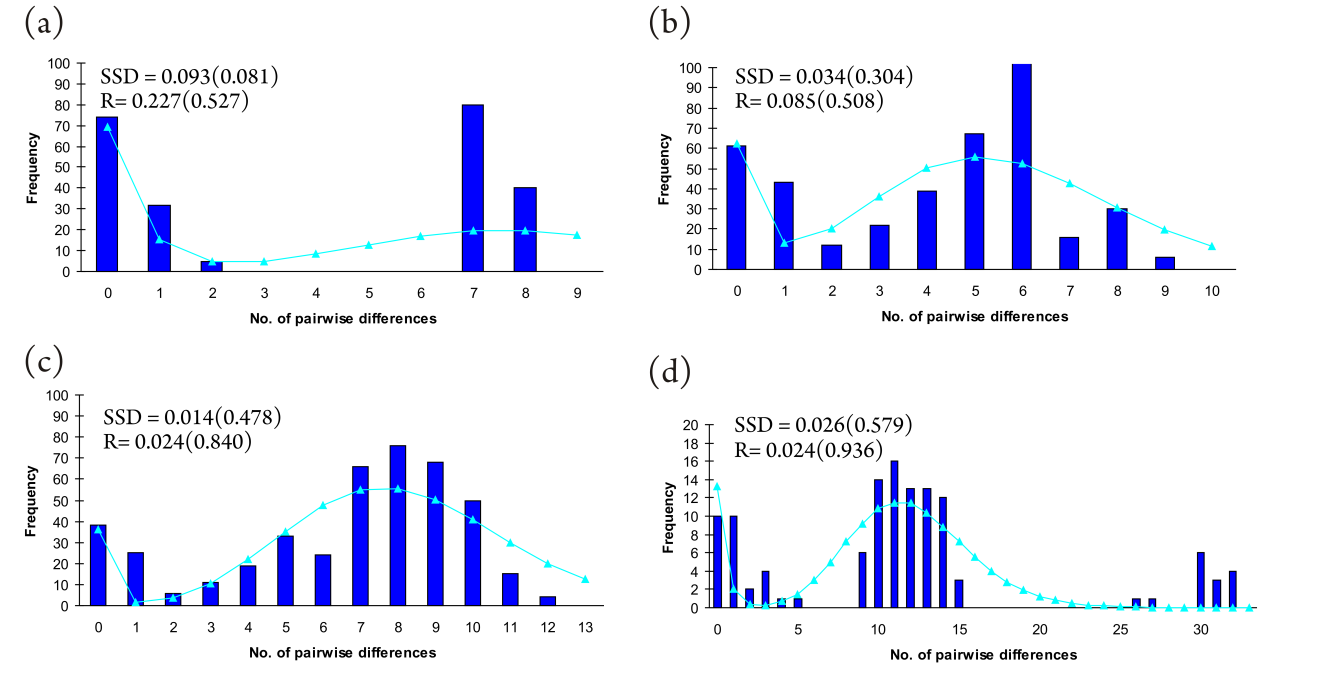


Figure S3. Principal component analysis (PCA) of the 1% of simulated datasets generated in DIYABC that were closed to the observed dataset in terms of summary statistics (see Methods) for the most likely colonisation scenarios (n=6) in analyses using mt and STR markers for *P. pacificus*, showing that most observed summary statistics fall within the range of simulated ones. Initial tests examined all possible orders (n=24) of island colonisation under the lineage diversification scenario U>D/A>C>B, and subsequent analysis considered the six most likely scenarios (see Table S3). The final most likely colonisation scenario (logistic regression value: 1.000) was: c>a>b>d. See Results for further information.


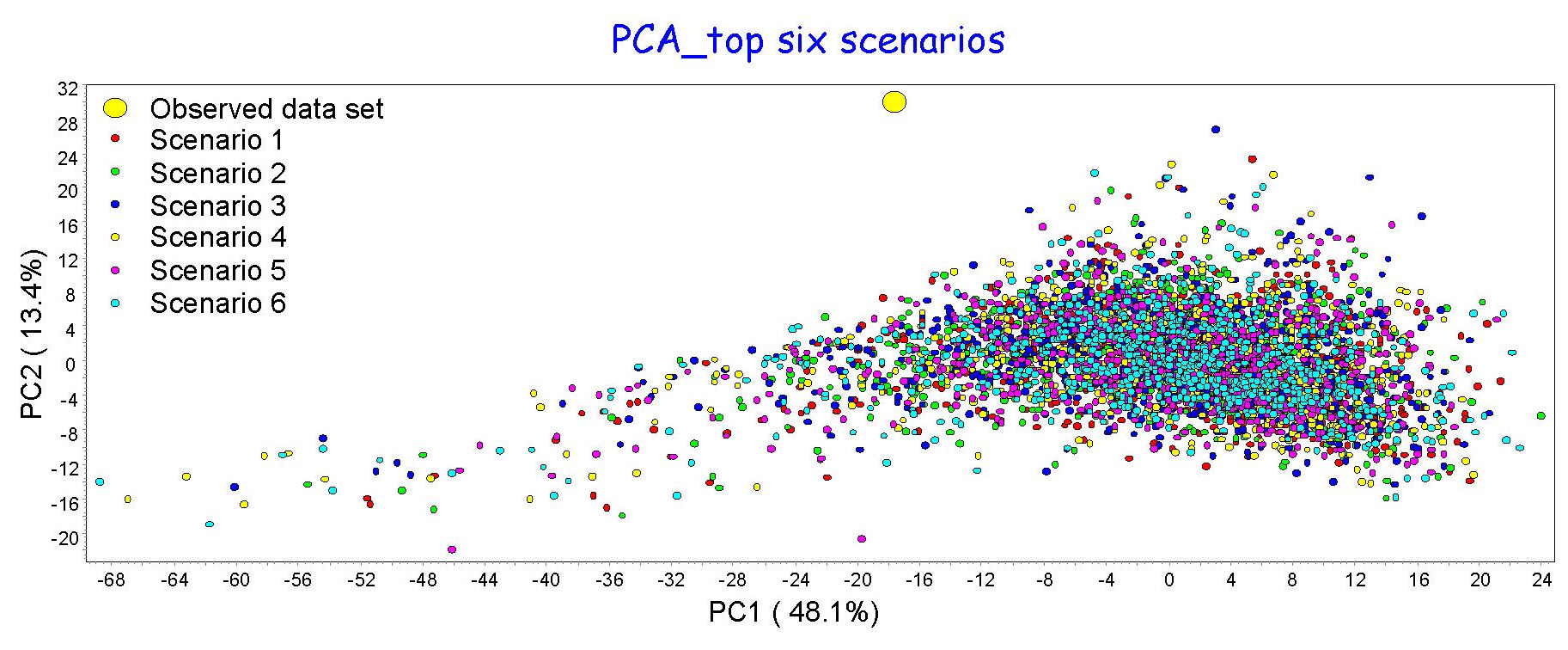

Supplement: Supplementary file 1 [file ece30003-0667-SD1.docx]
